# Analysis of differentially expressed genes in two immunologically distinct strains of *Eimeria maxima* using suppression subtractive hybridization and dot-blot hybridization

**DOI:** 10.1186/1756-3305-7-259

**Published:** 2014-06-03

**Authors:** Dandan Liu, Jianmei Li, Liqin Cao, Shangshang Wang, Hongxiao Han, Yantao Wu, Jianping Tao

**Affiliations:** 1 Jiangsu Co-innovation Center for Prevention and Control of Important Animal Infectious Diseases and Zoonoses, Ministry of Education Key Lab for Avian Preventive Medicine, Key Lab of Jiangsu Preventive Veterinary Medicine, College of Veterinary Medicine, Yangzhou University, 12 East Wenhui Road, Yangzhou, Jiangsu 225009, PR China

**Keywords:** *E. maxima*, Differentially expressed genes, Suppression subtractive hybridization (SSH), Dot-blot hybridization

## Abstract

**Background:**

It is well known that different *Eimeria maxima* strains exhibit significant antigenic variation. However, the genetic basis of these phenotypes remains unclear.

**Methods:**

Total RNA and mRNA were isolated from unsporulated oocysts of *E. maxima* strains SH and NT, which were found to have significant differences in immunogenicity in our previous research. Two subtractive cDNA libraries were constructed using suppression subtractive hybridization (SSH) and specific genes were further analyzed by dot-blot hybridization and qRT-PCR analysis.

**Results:**

A total of 561 clones were selected from both cDNA libraries and the length of the inserted fragments was 0.25–1.0 kb. Dot-blot hybridization revealed a total of 86 differentially expressed clones (63 from strain SH and 23 from strain NT). Nucleotide sequencing analysis of these clones revealed ten specific contigs (six from strain SH and four from strain NT). Further analysis found that six contigs from strain SH and three from strain NT shared significant identities with previously reported proteins, and one contig was presumed to be novel. The specific differentially expressed genes were finally verified by RT-PCR and qRT-PCR analyses.

**Conclusions:**

The data presented here suggest that specific genes identified between the two strains may be important molecules in the immunogenicity of *E. maxima* that may present potential new drug targets or vaccine candidates for coccidiosis.

## Background

Coccidiosis is an economically important disease that results in severe losses to the poultry industry and causes annual production losses estimated at $3 billion worldwide because of decreased feed conversion and high morbidity [1]. Coccidiosis is caused by several species of *Eimeria* apicomplexan protozoa, which colonize the intestinal mucosa [1] and current control methods rely mostly on the use of chemoprophylaxis or attenuated vaccine strains [2,3]. The induced immune response following infection with avian *Eimeria* is species-specific; therefore, the most frequently used live vaccines, such as “Coccivac”, “Immucox”, “Livacox,” and “Paracox”, should include common pathogenic species and strains that affect poultry [4-7].

Of the seven species that infect chickens, *E. tenella*, *E. maxima*, and *E. acervulina* are considered the most economically relevant [8]. *E. maxima* is the most immunogenic of the seven *Eimeria* species [9] and infection with as few as five sporulated oocysts can induce long-lived sterile protective immunity [10]. However, *E. maxima* strains have the most significant antigenic variation [11-13], thus, as a result of this immunological variability, vaccination with a given suspension of live oocysts may not confer effective protection against field strains in dissimilar geographical locations. In fact, the *E. maxima* strain present in the “Immucox” vaccine does not always elicit sufficient immunity to challenge with heterologous strains of this species in the field [14]. Similarly, an assessment of reductions in oocyst output showed that a single infection with the *E. maxima* strain isolated from the “Coccivac” vaccine afforded 20.09–82.44% protection against challenges with ten *E. maxima* strains isolated from various geographic regions of China, and the reductions of oocyst output were greater than 75% for only three strains [15].

Despite the first report on immunological variability of *E. maxima* in 1974 [16], the genetic basis to this phenotype remains unknown. Barta et al. [11] analyzed infraspecific variations among five North American *E. maxima* strains (USDA 68, Guelph, Maryland, North Carolina, and Florida) and reported no strain-specific differences in the protein profiles of sporozoites using one dimensional polyacrylamide gel electrophoresis (PAGE). Using the mRNA differential display technique, Basak et al. [17] identified mRNA corresponding to the 453-bp complementary DNA (cDNA) fragment GS-453, which is expressed only in the Guelph strain, but not the sporocyst-derived M6 strain from Florida. GS-453 gene is a sporozoite gene and expressed during the earliest stages of oocyst sporulation and is continuously expressed up to and including in the excysted sporozoite. However, the reason for the differential expression of this gene between the two stains remains unknown. Also, it is unclear whether this gene is in any way responsible for the lack of cross-protection between these two strains.

The *E. maxima* Shanghai (SH) and Nantong (NT) strains were isolated from litter samples collected in commercial broiler houses in Shanghai and Nantong, China, respectively, and confirmed to be *E. maxima* by microscopic examination, as well as isoenzyme and sequence analyses of the internal transcribed spacer regions [17-20]. The extent of immunological cross-protection among the SH and NT strains and four other *E. maxima* strains isolated in China (Yangzhou, Fengyang, Longyan, and Guangzhou) showed that the SH strain conferred immunity only to homologous strains, where the NT strain conferred immunity against both homologous and heterogeneous strains [15]. However, no detectable strain-specific differences were observed in the protein profiles of sporulated oocysts using sodium dodecyl sulfate-polyacrylamide gel electrophoresis (SDS-PAGE) [21]. In this study, in order to elucidate the molecular basis of immunological variability among *E. maxima* strains, we investigated whether there were strain-specific differences in gene expression profiles between the NT and SH strains using the suppression subtractive hybridization (SSH) technique combined with dot-blot hybridization and quantitative real-time polymerase chain reaction (qRT-PCR) analysis.

## Methods

### Parasites and animals

The *E. maxima* SH and NT strains used in this study were isolated from litter samples collected in commercial broiler houses in 2001 in Shanghai and Nantong in Jiangsu Province, China, respectively, and maintained in our laboratory.

Suqiu Yellow chickens were used to obtain oocysts of both *E. maxima* strains. One-day-old chicks were obtained from the Poultry Institute, Chinese Academy of Agricultural Sciences (Yangzhou, China), reared in a coccidia-free isolation facility, and allowed unlimited access to water and food that did not contain any anticoccidial drugs or antibiotics. Chickens were orally inoculated with 5 × 10^4^ sporulated oocysts at 2 weeks of age. Feces were collected from days 6 to 9 postinfection. The methods for handling the parasites, preparation of infection doses, and detecting and recovering oocysts from infected chickens have been fully described elsewhere [22]. The unsporulated oocysts were purified by centrifugation, salt flotation, and treatment with sodium hypochlorite as previously described [23]. The purified unsporulated oocysts were stored at −70°C for further use.

All animal care and procedures were conducted in accordance with the guidelines for animal use in toxicology. The study protocol was approved by the Animal Care and Use Committee of the College of Veterinary Medicine, Yangzhou University.

### Total RNA and mRNA extraction

Approximately 2 × 10^6^ unsporulated oocysts of *E. maxima* strains SH and NT were quickly ground into powder in liquid nitrogen with a mortar and pestle prior to adding 3 mL of TRIzol reagent (Invitrogen, Carlsbad, CA, USA) for total RNA isolation, which was performed according to the manufacturer’s instructions. After the integrity of the total RNA was tested by 1.0% agarose gels, mRNA was isolated using the EZ Spin Column mRNA purification kit (Bio Basic Inc, Toronto, Canada) following the manufacturer’s recommended protocol. The purified mRNA was electrophoresed prior to quantification with an ultraviolet (UV) spectrophotometer (NanoDrop2000; Thermo Scientific, Waltham, MA, USA), and stored at −70°C until use.

### Construction of subtracted cDNA libraries using the SSH technique

Two subtractive cDNA libraries were prepared. The forward library (named the NT subtractive cDNA library) was prepared using NT strain mRNA as the tester and the SH strain mRNA as the driver. The reverse library (named the SH subtractive cDNA library) was prepared using SH strain mRNA as the tester and NT strain mRNA as the driver. The subtractive cDNA libraries were constructed using the PCR-Select cDNA subtraction kit (Clontech Laboratories, Inc., Mountain View, CA, USA) based on the manufacturer’s instructions. Briefly, 2 μg of mRNA was used to synthesize double-stranded cDNA. After *Rsa*I digestion and ligation of adaptors, differentially expressed cDNAs were normalized and enriched through two rounds of hybridization and PCR amplification. The PCR products were purified using the DNA Fragment Purification Kit (TaKaRa Bio, Inc., Shinga, Japan) and then directly inserted into T/A clone vectors using the pGEM-T Easy Vector System (Promega Corp., Madison, WI, USA), transformed into chemically competent DH5α *Escherichia coli* cells (Invitrogen), and cultured on Luria broth media plates supplemented with ampicillin and X-Gal/isopropyl β-D-1-thiogalactopyranoside at 37°C overnight.

### Identification of the cDNA insert size by PCR

The white clones were randomly chosen from the transformation plates and then incubated in liquid medium supplemented with 100 μg/mL of ampicillin overnight at 37°C. The SSH cDNA clone inserts were amplified by PCR using nested primers 1 and 2R provided with the kit (Clontech Laboratories, Inc.) by 15 cycles of 94°C for 30 s, 68°C for 30 s, and 72°C for 1.5 min. A 5-μL aliquot of each PCR product was electrophoresed on 1.2% agarose gels. The positive clones confirmed by PCR were used for screening by dot-blot hybridization.

### Preparation of digoxigenin (DIG)-labeled cDNA probes

Two kinds of cDNA, subtractive and unsubtractive cDNA, were prepared before labeling. The subtractive cDNA consisted of the secondary SSH PCR products. The unsubtractive cDNA consisted of the PCR products produced using the following methods. The cDNA produced by reverse transcription of mRNA was digested with the *Rsa*I restriction enzyme and followed by adaptor ligation and then two rounds of PCR, in which the conditions were similar to the nested PCR in SSH, performed to generate a sufficient quantity of cDNA. Both types of PCR products were purified using the TaKaRa DNA Fragment Purification Kit (TaKaRa Bio, Inc.).

DIG-labeled cDNA probes were prepared using the DIG High Prime DNA Labeling and Detection Starter Kit I (Roche Applied Science, Indianapolis, IN, USA) according to the manufacturer’s instructions. Briefly, the cDNA was digested with *Rsa*I to remove the adaptor sequence, as reported previously [24,25], and then purified. A 1-μg aliquot of cDNA was labeled with DIG-11-2′-deoxyuridine 5″-triphosphate (Roche Applied Science) in a randomly primed DNA-labeling reaction as described by the manufacturer. The labeling efficiency of the cDNA was determined following the user manual (Roche Applied Science). DIG-labeled cDNA was quantitated by UV spectrophotometry (NanoDrop2000, Thermo). When in use, the probes were diluted to 50 ng/mL using DIG Easy Hyb hybridization buffer (10 mL/100 cm^2^ membrane; Roche Applied Science).

### Differential screening of cDNA libraries

A 1-μL aliquot of the PCR product from each positive clone of both the NT and SH strain subtractive cDNA libraries was blotted in duplicate onto two positively charged nylon membranes (Roche Applied Science) in a 0.25 cm^2^ area, respectively. The membranes were washed briefly in 2 × sodium chloride-sodium citrate (SSC) buffer and placed on Whatman 3MM gel blotting paper and incubated for 30 min at 120°C according to the manufacturer’s instructions. Two membranes of each subtractive cDNA library were prehybridized for 30 min at 42°C with DIG Easy Hyb hybridization buffer and then hybridized overnight at 42°C with the homologous subtractive cDNA probes or the heterologous unsubtractive cDNA probes. The next day, the membranes were washed with 2 × SSC, 0.1% SDS at 25°C for 5 min (×2), and then with 0.5 × SSC, 0.1% SDS at 68°C for 15 min (×2). Finally, immunological detection was performed using the anti-DIG-AP included in the kit according to the manufacturer’s instructions (Roche Applied Science). The results from the two hybridizations were compared for each clone by UVP with PDQuest 7.4 2-D analytical software (Bio-Rad, Hercules, CA, USA) and those showing the greatest differential expression were selected for sequencing.

### DNA sequencing and bioinformatic analysis

515 clones from the two subtractive cDNA libraries were screened by dot-blot hybridization and only those with apparent differential signals were sequenced by the Hua Da Genomic Company (Beijing, China). To obtain expressed sequence tags (ESTs), the vector and adaptor sequences were removed. The ESTs were assembled into consensus sequences (contigs) using Lasergene 7.0 software (http://www.dnastar.com/t-allproducts.aspx) and subjected to searches against a non-redundant database using the BlastX algorithm (https://blast.ncbi.nlm.nih.gov/). If there were no obvious results by the BlastX search, the EST sequences were searched for homology using the BlastN algorithm (https://blast.ncbi.nlm.nih.gov/) to presume the hypothetical proteins of ESTs.

### Validation of differentially expressed genes by qRT-PCR

According to sequencing results, ESTs were randomly selected for RT-PCR to verify their existence in unsporulated oocyst mRNA. Primers for the RT-PCR were designed using Primer5.0 primer design software (Table 1). The ESTs, which were verified by RT-PCR, were then randomly selected for qRT-PCR analysis. The *E. maxima* 18S ribosomal RNA gene (accession number: EF122251) was used as an internal control. The cDNA was synthesized using Moloney murine leukemia virus reverse transcriptase (Promega Corp.) and random primers. Primers (Table 2) for real-time PCR were 19–23 bp in length with melting temperatures of 55–57°C. The PCR products were 160–250 bp in length. Real-time PCR was performed using SYBR Green I Real MasterMix reagent (TaKaRa Bio, Inc.). PCR for each EST was performed in triplicate. Melting curves were used to assess the reliability of PCR analysis. All data were analyzed using the Mastercycler ep Realplex real-time PCR system (Eppendorf International, Hamburg, Germany).

**Table 1:** The primers and gene ID, used for RT-PCR analysis

**Gene ID**	**Forward primer (5′-3′)**	**Reverse primer (5′-3′)**
S36	ACACAGCGACCAGTCCTCT	GAA AGC CGT CTC CGA AGC
S229	CACCCGTGTTTCCATGAGAG	CGACAATTCCCATCG AAGAT
S231	GCCGTCGTCGCTTTAGATATG	CACACGGCATCCACTTCAG
S251	CATGTTGCAAAGTAGCACTGT	TGGCTGTCGAGCGAGAAT
N140	CTGCTTCACCCTTTGCAC	CCTCTCACCAATCATTCTCAC
N214	CCCTGCAAAGTCGGTCAAG	AGCAGCAGAGGCAGCGTTG

**Table 2:** The primers and gene ID, used for qRT-PCR analysis

** Gene ID**	**Forward primer (5′-3′)**	**Reverse primer (5′-3′)**
18 s rRNA	CTAACGCAAGGAAGTTTGAGGCA	TACAAGAGGCAGGGACGTAATCG
S36	CAACTCTGAAGCCTGAACTGGC	CTGTCATTGTGGACCTTGCAAC
S251	GTCCAACCGCAATAACATCG	CACTTTCGCAACGGAACTCA
S229	ATTCAGGCACCCGCACTAT	TACGGCGGAACTATCACCAAT
S231	TAGGTTGCTGCTAACCATACAGG	CACACGGCATCCACTTCAG
N140	CTTGCACCGCACAGCATAATGT	TCGTCCTGAGTGGAGGCTTTCT
N214	ATAGTCGGGAATCCAACAACTG	GCCAACCCTACTGCTTGCTG

### Statistical analysis

Data were expressed as mean ± S.D. Statistical analysis were performed using the Duncan’s multiple range test with SPSS 13.0. Values of P < 0.05 were considered differences and P < 0.01 were considered significant differences.

## Results

### Construction of subtractive cDNA libraries

**Figure 1:**
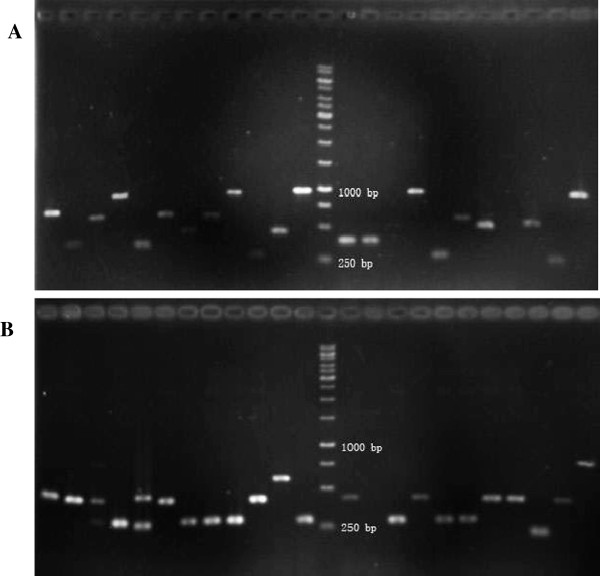
**Gel analysis of the inserted cDNA fragments in subtractive cDNA libraries of two strains of *Eimeria maxima*: SH (A) and NT (B) strain.**

The purified second round PCR products in SSH were ligated into the T/A clone vectors and then electrotransformed into competent DH5α *E. coli* cells. The positive clones were screened by the blue-white colony screening method. Finally, a total of 561 clones was randomly chosen from the two subtractive libraries (300 from the SH subtractive cDNA library and 261 from the NT subtractive cDNA library). On the basis of PCR analysis results with nested PCR primers 1 and 2R, the insert efficiency of the SH and NT subtractive cDNA library clones were nearly 95% (284/300) (Figure 1A) and 89% (231/261) (Figure 1B), respectively, and the insert fragments were mainly between 250–1000 bp.

### Differential screening of cDNA libraries by dot-blot analysis

**Figure 2:**
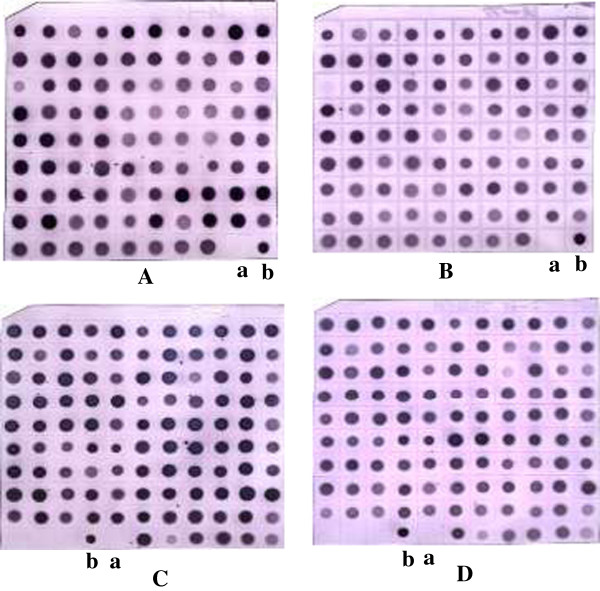
**Dot-blot hybridization, A and B was used to test the cDNA library of NT strain, C and D was used to test the cDNA library of SH strain. A**: The probes that came from subtractive PCR products of the NT strain were used to test the cDNA library of NT strain; **B**: The probes that came from unsubtractive PCR products of the SH strain were used to test the cDNA library of NT strain; **C**: The probes that came from subtractive PCR products of the SH strain were used to test the cDNA library of SH strain; **D**: The probes that came from unsubtractive PCR products of the NT strain were used to test the cDNA library of SH strain. Each clone was blotted in the same place in two identical membranes. a were negative control; b were positive control.

Based on PCR identification, 515 clones (284 and 231 from the SH and NT subtractive cDNA libraries, respectively) were screened by dot-blot hybridization. To exclude false-positive clones, two rounds of dot-blot hybridization were carried out. The positive clones identified in the first round were used for the second round of dot-blot hybridization. After the first round of dot-blot hybridization, a total of 192 positive clones were identified according to strong hybridization signals, including 104 clones from the SH subtractive cDNA library and 88 from the NT subtractive cDNA library. After the second round of dot-blot hybridization, a total of 86 differentially expressed clones were identified, including 63 from the SH subtractive cDNA library (Figure 2A and B) and 23 from the NT subtractive cDNA library (Figure 2C and D). These 86 clones were selected for sequencing and further analysis.

### cDNA sequencing

The 86 differentially expressed clones were sequenced and each produced high-quality sequences. The 86 ESTs were assembled into seven contigs and three singlets using Lasergene 7.0 software, resulting in ten assembled sequences. Of these, six sequences were obtained from the SH subtractive cDNA library and four from the NT subtractive cDNA library (Table 3). Each assembled sequence was subjected to BlastX and BlastN searches to identify homologous sequences deposited in the National Center for Biotechnology Information (NCBI) database (http://www.ncbi.nlm.nih.gov/) (Table 4). After performing the Blast searches, all six contigs from the SH strain matched homologous sequences with identities of 31–92%, which have similarity with either transmembrane domain-containing proteins or hypothetical proteins of *Toxoplasma gondii* ME49, aminopeptidase-like proteins of *Thalassiosira pseudonana*, *Plasmodium falciparum* isolate merozoite surface proteins, or hypothetical proteins of *Plasmodium vivax* and *T. gondii*. Three of the four contigs from the NT strain matched homologous sequences with identities of 65–83%, which included the BT1 transmembrane domain-containing protein of *T. gondii* ME49, a hypothetical protein of *E. tenella*, and myosin light chain kinase (MLCK) of *E. tenella*. There were no matches for one contig from each library, which were presumed to be novel sequences. All of the ESTs reported in this study were submitted to the NCBI dbEST database and assigned the GenBank accession numbers JK823412–JK823421.

**Table 3:** Primary analysis of the contigs of the sequences from subtractive library

**Library name**	**Contigs**	**Length of sequence (bp)**	**Sequences**
SH strain	contig 1	985	53
	contig 2	720	4
	contig 3	334	2
	contig 4	384	2
	contig 5	841	1
	contig 6	458	1
	total of 6 contigs	-	Total 63 sequences
NT strain	contig 1	737	10
	contig 2	383	7
	contig 3	413	5
	contig 4	543	1
	Total of 4 contigs	-	Total of 23 sequences

**Table 4:** Primary homologous analysis of the genes filtered from subtractive library

**Clone ID**	**Frequency**	**Homologous gene**	**Accession number**	**Identity (%)**	**Program**
N140	7	*Toxoplasma gondii* ME49 BT1 transmembrane domain-containing protein	XP_002368033.1	81%	BlastX
N144	5	*Eimeria tenella* hypothetical protein	XP_001238682.1	83%	BlastX
N199	1	*Eimeria tenella* myosin light chain kinase partial mRNA	XM 001238742.1	65%	BlastN
N214	10	-	-	-	-
S36	2	*Eimeria acervulina* 1A protein	ADQ44148.1	92%	BlastX
S189	2	Hypothetical protein TGME49-059590 [*Toxoplasma gondii* ME49]	XP 002365153.1	55%	BlastX
S226	4	Aminopeptidase-like protein [*Thalassiosira pseudonana* CCMP 1335]	XP 002296782.1	50%	BlastX
S229	1	Hypothetical protein TGGT1 236900 [*Toxoplasma gondii* GT1]	EPR60247.1	31%	BlastX
S231	1	*Plasmodium falciparum* isolate 7G8 merozoite surface protein 9 gene, partial CDS	FJ406825.1	81%	BlastN
S251	53	Hypothetical protein [*Plasmodium vivax* SaI-1]	XP 001615098.1	45%	BlastX

### Real-time validation of the genes identified by SSH

**Figure 3:**
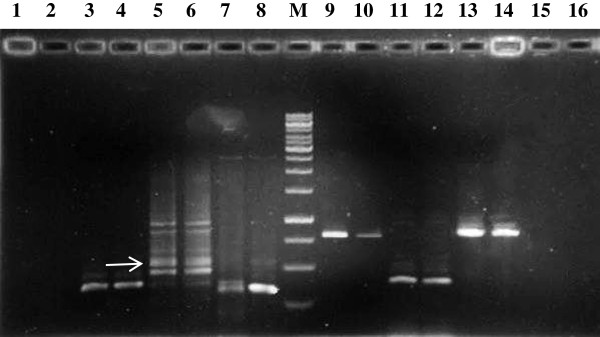
**Gel analysis of the differentially expressed gene filtered from the SSH library.** Lane M: 1 kb DNA ladder; lane 1, 3, 5, 7, 9, 11, 13 used NT strain cDNA as template; lane 2, 4, 6, 8, 10, 12, 14 used SH strain cDNA as template. Lanes 1 and 2 were negative control without reverse transcription. Lanes 15 and 16 were no template control. Line 3 and 4, 5 and 6, 7 and 8, 9 and 10, 11 and 12, 13 and 14 used specific primers of N140, N214, S36, S231, S229, S251, respectively. The arrow shows the aimed RT-PCR products.

**Figure 4:**
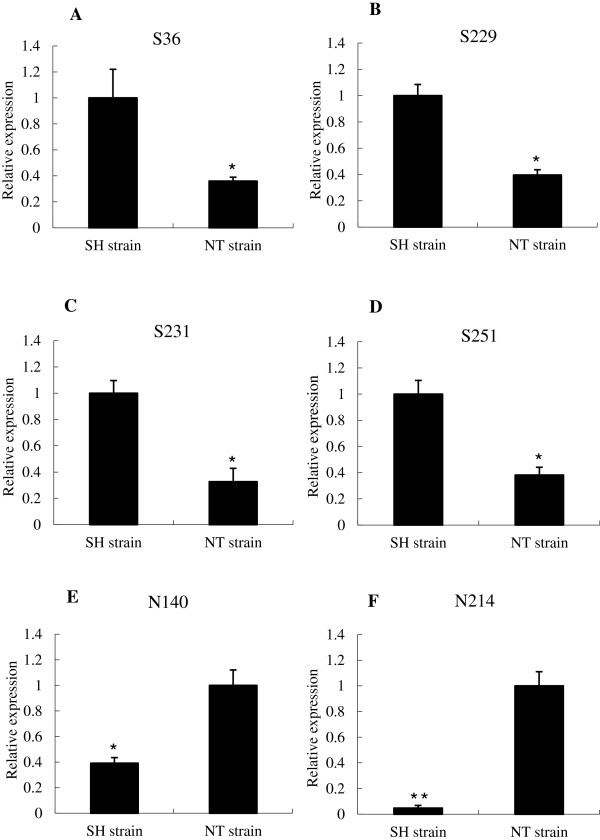
**Relative expression of sequences in SH and NT strain by qRT-PCR analysis.** The results of the relative expression of S36, S229, S231, S251 were shown as **A**, **B**, **C** and **D**, N140 and N214 were shown as **E** and **F**. Each bar represents the mean ± S.D. value (n = 3). *, P < 0.05; **, P < 0.01.

To verify that genes identified by SSH combined with dot-blot hybridization were differentially expressed in the SH and NT strains, real-time PCR was performed to validate the results. At first, six of ten contigs (S36, S229, S231 S251, N140, and N214) were selected for reverse transcription PCR to detect their existence (Figure 3). For every primer pair, we used the same RNA preparation without reverse transcription (−RT control) and a no template control for analysis, the results was negative. Then, each was selected for further real-time PCR analysis. The amplification efficacies of aimed genes and reference genes ranged from 95% to 102% (Table 5). The expression levels of the four unique sequences (S36, S229, S231, and S251) were upregulated in strain SH and downregulated in strain NT (Figure 4A–D), and expression of two sequences (N140 and N214) were upregulated in strain NT (Figure 4E and F).

**Table 5:** The amplification efficiency of reference gene and aimed genes

**Gene ID**	**Slope**	**Y-intercept**	**Efficiency**	**R**^**2**^
18 s rRNA	−3.27	34.78	1.02	0.9926
S36	−3.44	46.26	0.95	0.9884
S251	−3.35	47.05	0.98	0.9836
S229	−3.27	48.12	1.02	0.9819
S231	−3.35	47.05	0.99	0.9836
N140	−3.29	34.78	1.01	0.9871
N214	−3.34	34.45	0.99	0.9828

## Discussion

Antigenic diversity is a feature of many classes of pathogens, including viruses, bacteria, protozoa, and helminthes. There are mainly two types of mechanisms mediating antigenic variability. The first arises by switching between variant proteins encoded by large polymorphic gene families, whereas the second involves genetic diversity within the pathogen [26]. The mechanisms mediating antigenic variation in *E. maxima* involve genetic diversity [16,27]. The antigenicity of *E. maxima* strains appears to be stable with time [28], but this stability is distinct from the dynamic process of antigenic variation that occurs with other protozoans [26]. A previous study confirmed that a larger number of loci likely contributes to strain-specific immunity [29]. Furthermore, the *Eimeria* life-cycle is largely haploid [30] and, theoretically, any of the allelic genes can be expressed. Therefore, the genes related to antigenic variation between two immunologically distinct strains of *Eimeria* can be identified using methods for cloning differentially expressed genes. Using the mRNA differential display technique, Basak et al. [17] isolated and cloned a 453-bp cDNA fragment from the *E. maxima* Guelph strain, but not the Florida strain.

In the present study, a total of 86 differential ESTs were obtained from 515 clones, 63 from strain SH and 23 from strain NT. After sequencing and bioinformatic analysis, ten contigs were obtained, six from strain SH and four from strain NT. Real-time PCR was used to validate the reliability of the results. By comparing these ten contigs with previous dbESTs deposited in the NCBI database, all six SH strain contigs and three of the four from the NT strain shared significant identity with previously described or hypothetical proteins, which were present in the sequenced genomes of all apicomplexan parasites, including *Eimeria spp.*, *T. gondii*, *Theileria spp.*, *and Plasmodium spp.*

Some homologous proteins that have been implicated in acute infection are transmembrane proteins [*T. gondii* ME49 (TGME49) and TGGT1], which can enhance the ability of parasites to invade after extracellular stress [31,32]. These transmembrane proteins are required for survival of *T. gondii* during infection, promotion of extracellular survival, and enhanced penetration after extracellular stress [33,34]. Each was involved in replication in activated immune cells and the establishment of chronic infection.

Another homologous protein identified was the MLCK of *E. tenella*, which is actively involved in the contraction of epithelial perijunctional actinomyosin rings, thereby increasing paracellular permeability [35]. Also, MLCK plays a role in alterations of epithelial barrier function in *Giardia* spp. [36]. Thus, this protein may enhance the penetration of the host cell, thereby facilitating invasion. Similarly, the *Plasmodium falciparum* merozoite surface protein is a target of allele-specific immunity and alleles are maintained by natural selection. Moreover, the merozoite surface protein plays an essential role in the merozoite invasion of red blood cells.

The *Thalassiosira pseudonana* aminopeptidase-like protein (CCMP 1335) appears to be similar in function to *P. falciparum* aspartyl aminopeptidase, which is expressed in the cytosol of the parasite and exported to the parasitophorous vacuole, indicating that this enzyme may have a function outside the parasite [37] and in concert with protein catabolism [38-40]. This process includes breakdown and turnover of the *Plasmodium* cellular proteins for the anabolism of proteins required for the rapidly growing intracellular parasite.

The *E. acervulina* 1A protein, also named the *Eimeria* refractile body protein, is an invasion-related protein in sporozoites and first generation merozoites. Ea1A may store specific nutrients that are essential during the passage through the gut and the first hours of intracellular development [41]. Also, Ea1A may contain pyridine nucleotide transhydrogenase, which is a redox enzyme for direct catalyzation of the reversible hydride transfer between nicotinamide adenine dinucleotide phosphate and nicotinamide adenine dinucleotide, which regulates and restores homeostasis of these two redox cofactors in catabolism and anabolism. This procedure can also enhance sporozoitic invasion [42,43].

In this study, some differentially expressed genes were found to encode proteins of unknown function, but homologous proteins were identified in other apicomplexan parasites*.* Further characterization of these genes and their products will provide useful information to identify genes responsible for the immunological variability of *E. maxima*.

According to the results of our real-time PCR analysis*,* the genes in homologous strains were expressed on a higher level. However, the reasons that expression of some genes were on a higher level in one *E. maxima* strain and not in the other remains unknown, nor was it clear if this gene was in any way involved in the lack of cross-protection between these two strains. The function and localization of the differentially expressed genes warrant further study to provide important background information regarding the molecular mechanisms underlying differences in immunogenicity and in molecular evolutionary patterns of coccidians.

## Conclusion

In conclusion, ten specific genes were identified between the antigenically distinct *E. maxima* SH and NT strains*.* These distinctive differentially expressed genes may play important roles in development, growth, metabolism, and proliferation. Further characterization of these distinct genes will provide useful information to further elucidate antigenic polymorphisms.

## Competing interests

The authors declare that they have no competing interests.

## Authors’ contributions

DD and JP conceived and designed the study, and critically revised the manuscript. DD, JM and LQ performed the experiments, analyzed the data and drafted the manuscript. SS, HX and YT helped in the study design. All authors read and approved the final manuscript.
